# Prenatal exposure to antihypertensive medication: A systematic review of neurodevelopmental and educational outcomes

**DOI:** 10.1002/jcv2.70080

**Published:** 2025-12-19

**Authors:** Shrifah Alkhalaf, Sarjit Singh, Jill P. Pell, Scott M. Nelson, Daniel F. Mackay, Michael Fleming

**Affiliations:** ^1^ School of Health and Wellbeing University of Glasgow Glasgow UK; ^2^ School of Medicine Dentistry and Nursing University of Glasgow Glasgow UK

**Keywords:** antihypertensive, child development, education, neurodevelopment, prenatal

## Abstract

**Background:**

Where possible, medication is avoided during pregnancy as it can cross the placenta and potentially cause congenital malformations, impair growth and have longer‐term functional or cognitive sequelae. However, medication is sometimes required for pre‐existing or new‐onset conditions such as hypertension disorders during pregnancy; the longer‐term outcomes of which remain unclear. This systematic review aimed to synthesise the evidence on prenatal exposure to antihypertensive medication and longer‐term neurodevelopmental and educational outcomes up to 18 years of age.

**Methods:**

We searched Medline Ovid, PubMed, Excerpta Medica Database, and Web of Science from inception (1946) until 04 July 2025, selecting observational and experimental studies. The inclusion criteria for eligible studies were pregnant women with or without any hypertension disorder of pregnancy, mothers taking antihypertensive medication during pregnancy comparing them with those not exposed, children ≥1 years of age, and children with one of the predefined neurodevelopmental and educational outcomes. The studies' quality was evaluated using the National Heart, Lung, and Blood Institute assessment tool.

**Results:**

Of 11 studies included (five cohorts, two case‐control and four randomised controlled trials), 10 were of high quality, while only one was of low quality. Six of the 11 studies demonstrated significant associations between prenatal exposure to antihypertensive medication and at least one adverse neurodevelopmental outcome in children. One study reported an increased risk of autism spectrum disorder following calcium channel blockers and beta‐blockers exposure, one reported the risk of fine motor problems following calcium channel blockers exposure, one reported increased risk of low intelligence quotient following exposure to methyldopa, one reported an increased risk of attention deficit hyperactivity disorder following exposure to beta‐blockers, one reported speech problems following exposure to more than one antihypertensive medications, and one reported an increased risk of sleeping disturbance following exposure to clonidine. Methodological heterogeneity, small sample sizes, and inadequate confounder adjustment were common limitations.

**Conclusions:**

The association between prenatal antihypertensive exposure and neurodevelopmental outcomes warrants further research, focusing on the impacts of specific medication classes on a range of outcomes in the same study population.

## Key Points

**What's known?**

- Hypertension has adverse effects on mothers and their children's health.
- Treatment is required; however, medication is known to adversely affect pregnancy and neonatal outcomes.
- The existing evidence is limited on longer‐term neurodevelopmental child outcomes but suggests that prenatal exposure to antihypertensive medication may be associated with adverse neurodevelopmental outcomes.

**What's new?**

- Understanding potential risks of prenatal exposure to antihypertensive medication is essential to informed decision‐making.

**What's relevant?**

- Research findings will help to enhance maternal awareness and prevent theses outcomes in the future.

## Graphical abstract

This systematic review investigated the association of prenatal exposure to antihypertensive medication with longer‐term poor neurodevelopmental outcomes by evaluating the findings from existing literature. We undertook a systematic review of previous studies looking specifically at neurodevelopmental outcomes in children. The existing evidence is limited but suggests that prenatal exposure to antihypertensive medication may be associated with adverse neurodevelopmental outcomes.

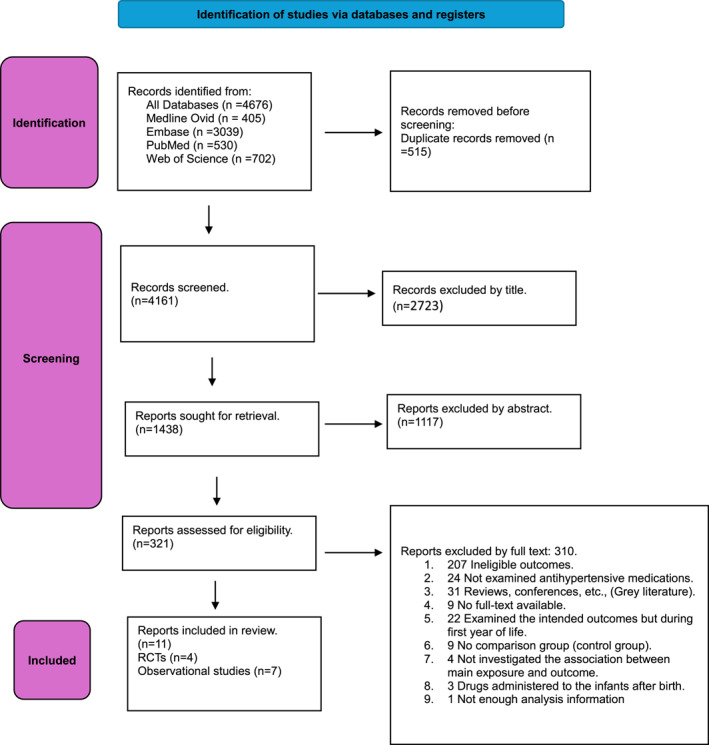

## INTRODUCTION

Hypertensive disease during pregnancy includes chronic hypertension, gestational hypertension, preeclampsia, and chronic hypertension with superimposed preeclampsia. Between 1990 and 2019, there has been a 10.9% rise in the global incidence of hypertensive disorders of pregnancy (HDP); from 16.30 million cases per annum to 18.08 million cases per annum (Wang et al., 2021). Despite general avoidance of medication during pregnancy, anti‐hypertensives drugs remain crucial for controlling pregnancy‐related hypertension and minimising maternal and foetal morbidity and mortality (Fitton et al., 2017).

Recent research has increasingly highlighted the potential link between hypertensive disorders in pregnancy, such as preeclampsia, and adverse neurodevelopmental outcomes in children. A 2016 systematic review firmly established a connection between preeclampsia and reduced cognitive function in offspring (Pinheiro et al., 2016). Subsequent cohort studies have expanded this understanding, revealing associations with neuropsychiatric disorders, autism spectrum disorder (ASD), attention‐deficit/hyperactivity disorder (ADHD), and intellectual disabilities (Brand et al., 2021; Nahum Sacks et al., 2019). However, distinguishing the effects of hypertensive disease itself from the effects of antihypertensive medication remains a complex and unresolved issue.

Hypertensive disorders during pregnancy are associated with a range of longer‐term adverse neurodevelopmental and educational child outcomes. Childhood neurodevelopmental problems caused by disruption of the development of the brain and nervous system can lead to cognitive, motor, language, behavioural and social‐emotional problems (Chen et al., 2021). These can present as a very wide range of clinical conditions including cerebral palsy, developmental delays, ASD, ADHD, and intellectual disabilities (Thapar et al., 2017). These outcomes are shaped by both genetic and prenatal environmental factors and can be diagnosed at various stages from early foetal life until early adulthood (Saudubray & Garcia‐Cazorla, 2018). Educational difficulties impact a child's ability to learn which significantly affects academic performance, psychosocial wellbeing, and concentration levels, and can cause physical symptoms (Children with Special Educational Needs, 2012). Educational outcomes can be measured through academic achievement, academic progress, and a record of special educational support needs.

In the last three decades, 80%–90% of pregnant women have taken at least one medication during pregnancy, including over the counter medications. Since many pregnancies are unplanned, over 80% of these women, take the medication during the first trimester, a critical period for foetal neurodevelopment and organogenesis (Haas et al., 2018; Lupattelli et al., 2020; Thorpe et al., 2013). Exposure to medication during the first trimester can affect normal cell signalling, proliferation, and differentiation that can lead to congenital anomalies or miscarriage (Buawangpong et al., 2020). This stage of embryo development determines susceptibility to teratogens where functional disturbances are likely to occur due to developing organs being sensitive to teratogenic effects during rapid cell division and differentiation (Orna, 2011). A meta‐analysis found that prenatal exposure to angiotensin‐converting enzyme (ACE) inhibitors or angiotensin receptor blockers in the first trimester was significantly associated with increased risk of cardiovascular malformations, miscarriages, and stillbirths (Buawangpong et al., 2020). Use of medication during the second and third trimesters can also affect behavioural and functional development of the foetus (Fitton et al., 2017).

Due to ethical reasons, clinical trials of medication safety are rarely conducted on pregnant women. Most medication has not been tested during human pregnancy and animal models may not accurately reflect effects in humans (Adam et al., 2011). Thus, understanding is lacking on the clinical and public health implications of antenatal medication use (Lupattelli et al., 2020). While medication is generally avoided in pregnancy due to potential risks to the foetus, it may be essential for treating maternal conditions that, if left untreated, could pose greater harm to both mother and child (Caton et al., 2009). Hence, understanding and quantifying the risks versus benefits is important.

The understanding of prenatal exposure to antihypertensive medication and its effects remains limited. While there is evidence linking such exposure to adverse perinatal outcomes, including Apgar score components (poor appearance, pulse, grimace, activity, and respiration) (Koren, 2013) and higher risk of congenital malformations (Fitton et al., 2017), the data regarding long‐term neurodevelopmental impacts are inconsistent (Fitton et al., 2017). Some studies suggest that exposure during the second or third trimester may be associated with long‐term functional and behavioural abnormalities (Fitton et al., 2017), but other studies show no association with general health, and vision and hearing problems and even reduced likelihood of delayed fine‐motor function at 18 months of age compared with untreated hypertension (Koren, 2013). The aim of this systematic review is to consolidate the published evidence on the associations between prenatal exposure to antihypertensive medication and neurodevelopmental and educational outcomes up to ≤18 years of age.

## MATERIALS AND METHODS

The review was conducted in accordance with Preferred Reporting Items for Systematic Reviews and Meta‐Analyses standards (PRISMA, 2020). We conducted a comprehensive search in Medline Ovid, Medline Excerpta Medica Database, PubMed, and Web of Science, from their inception (1946) to 04 July 2025. Our focus was on the impact of prenatal antihypertensive medication exposure on neurodevelopmental and educational outcomes. The research questions and study selection were guided by the Population, Intervention, Comparator and Outcomes framework (Stern et al., 2014).

**FIGURE 1:**
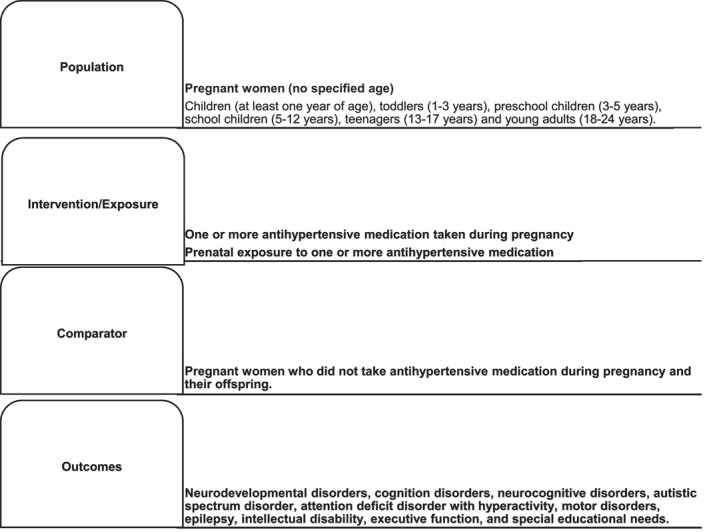
The Population, Intervention, Comparator and Outcomes framework.

The population was pregnant women of no specified age with or without any hypertension disorder of pregnancy and their offspring including: children (at least 1 year of age), toddlers (1–3 years), preschool children (3–5 years), school children (5–12 years), teenagers (13–17 years) and young adults (≤18 years). The exposure was defined as one or more antihypertensive medication taken during pregnancy. Studies were included if they compared pregnant women with the exposure to pregnant women without the exposure. The outcomes of interest were defined as: neurodevelopmental disorders, cognition disorders, neurocognitive disorders, ASD, ADHD, motor disorders, epilepsy, intellectual disability, executive function, and special educational needs (Figure 1). Full details of the search strategy are provided in the (Appendix S1).

Two independent reviewers (S.A. and S.S.) screened studies using predefined criteria. We included both observational and experimental studies—prospective and retrospective cohort studies, cross‐sectional studies, case‐control studies, and trials—conducted on humans and published in English. The review excluded unpublished studies (grey literature) and studies of maternal, pregnancy or perinatal outcomes.

Quality assessment was conducted using the National Heart, Lung, and Blood Institute (NHLBI) tool (Study Quality Assessment Tools) which evaluates the study quality, strength of evidence and risk of embedded biases. This tool was developed by NHLBI in 2013 and was administered in accordance with the instructions provided by the National Institutes of Health. It is one of the validated quality assessment tools that evaluates both internal validity and risk of bias. Risk of bias was evaluated by the two reviewers independently and divergent views resolved through discussion. This tool, adaptable for various study designs and used to update clinical guidelines, scores studies based on 14 criteria with higher scores indicating better quality. Studies scoring ≥8 were categorised as good, 6–7 as fair, and ≤5 as poor.

## RESULTS

We extracted the following data from the studies: year, study design, sample size, participant demographics, exposure details (e.g., type of antihypertensive medication), and outcomes assessed. These data were tabulated and grouped by class of antihypertensive medication: beta‐blockers, calcium channel blockers, diuretics, methyldopa, ACE inhibitors, and angiotensin receptor blockers.

### Study characteristics

**FIGURE 2:**
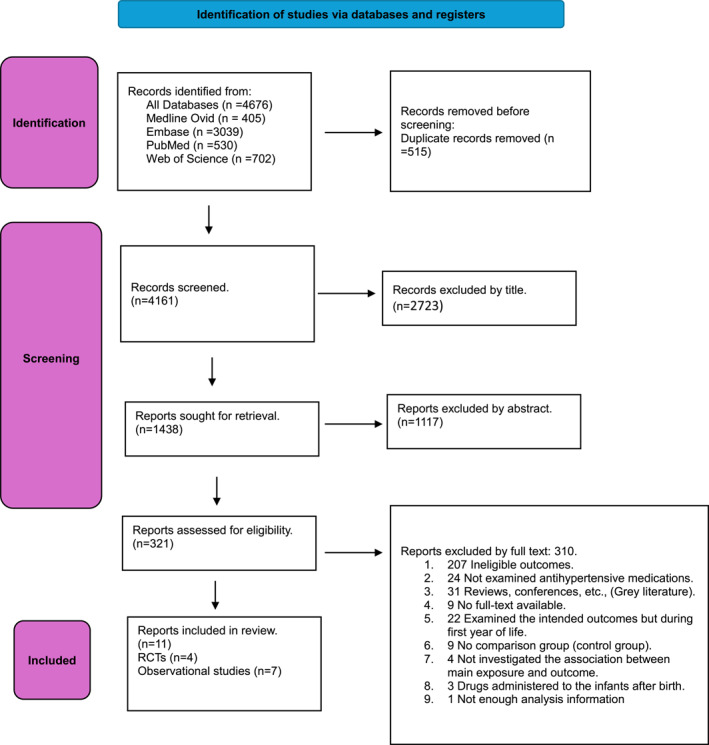
Preferred Reporting Items for Systematic Reviews and Meta‐Analysis 2020 flow chart of articles identified, screened, and included.

From 4161 articles initially identified following removal of duplicates, 11 were eligible for inclusion in the review (Figure 2): four investigating calcium channel blockers, two investigating beta‐blockers and methyldopa, two investigating unspecified antihypertensive drugs (mainly methyldopa), one investigating the alpha agonist clonidine, one investigating beta‐blockers and calcium channel blockers, and one investigating more than one antihypertensive drug. Of these, five were cohort studies, two case‐control and four randomised control trials (RCTs). The age of the children investigated in the included studies ranged from 18 months to ≤18 years and sample sizes of these studies varied significantly, ranging from 44 to 117,963 participants, with the majority (*n* = 8) published post‐2000 (Table 1). Geographically, the studies spanned Europe, Canada, the USA, and UK.

**TABLE 1:** Characteristics of eligible studies.

First author	Year	Country	Size	Study design	Participants underlying condition status	Comparison group	Exposure or intervention	Exposure measurement	Outcomes	Outcome measurement	Age at outcome
1. Cockburn et al. (1982)	1982	UK	195	RCT	Mild and moderate hypertension during pregnancy	Untreated hypertensive women	Specific antihypertensive treatment (mainly methyldopa)	Intervention	Copying, verbal tactile matching, delayed visual recall, immediate visual recall, recall of designs, recall of digits, visual recognition, basic arithmetic word definitions, verbal fluency, reading quotient	British ability scales; Holborn reading test	7½ years
2. Bortolus et al. (2000)	2000	Italy	190	RCT	Mild‐moderate hypertension in pregnancy	Untreated hypertensive women	Nifedipine	Intervention	Gross/fine motor, hearing/sight, language	Griffiths mental developmental scale	18 months
3. Fitton et al. (2021)	2020	UK	117,963	Retrospective cohort	Treated late‐onset hypertension (pre‐eclampsia) Untreated hypertension In‐utero exposure to antihypertensive medication (in the presence of hypertension)	Untreated hypertensive women	≥1 antihypertensive medication	Dispensing data	Gross/fine motor, vision, hearing, speech, attention, emotional, behavioural, social developmental problems	Ages and stages questionnaire	27–30‐months
4. Hisle‐Gorman et al. (2018)	2018	USA	8760 ASD; 26,280 no ASD	Case‐control	Treated hypertension disorders during pregnancy Untreated hypertension disorders during pregnancy In‐utero exposure to antihypertensive medication only (in the absence of hypertension)	Untreated hypertensive women	Calcium channel blockers, β‐blockers	Pharmaceutical records	ASD	International >1 ICD‐9 code	2–18 years
5. Pasker‐De Jong et al. (2010)	2010	Netherlands	202	Retrospective cohort	Mild‐to‐moderate gestational hypertension	Bed rest (normal/control) group	Labetalol; methyldopa	Hospital records	ADHD, sleep, IQ, concentration, gross/fine motor, visual/associative/auditory memory	Child behaviour checklist; teacher report form	4–10 years
6. van Vliet et al. (2015)	2015	Netherlands	170	RCT	Not specified	Placebo (normal/control) group	Nifedipine	Intervention	Communication, gross/fine motor, problem‐solving, personal–social functioning, developmental delay	Ages and stages questionnaire	2 years
7. Ounsted et al. (1980)	1980	UK	168	RCT	Hypertension in pregnancy (i.e. pre‐existing hypertension, gestational hypertension, pre‐eclampsia)	Untreated hypertensive women	Specific antihypertensive treatment (mainly methyldopa)	Intervention	Gross, fine, visuo motor function; language, comprehension	Sight and hearing tests, somatic measures, questionnaires, developmental assessment	4 years
8. Huisjes et al. (1986)	1986	Netherlands	44 children	Retrospective case‐control	Hypertension or superimposed preeclampsia	Untreated hypertensive women	Clonidine	Medical database	Neurological problem, school performance, behaviour, hyperactivity, sleep	Questionnaire	3–9 years
9. Chan et al. (2010)	2010	Canada	99 mother‐child pairs	Prospective cohort	Hypertension in pregnancy (i.e., pre‐existing hypertension, gestational hypertension, pre‐eclampsia)	Normotensive (normal/control) group	Labetalol; methyldopa	Standardized questionnaires	Full, performance and verbal IQ	Wechsler preschool and primary scale of intelligence	3–7 years
10. Lorthe et al. (2024)	2024	France	712	Prospective cohort study—population based	Pregnant women with preterm prelabour rupture of membranes	Not exposed to tocolysis	Calcium channel blockers (atosiban and nifedipine)	Medical records	Survival without moderate to severe neurodevelopmental disabilities survival without any neurodevelopmental disabilities	Standardized neurodevelopmental assessment scales	5.5 years
11. Plouchart et al. (2024)	2024	France	1320	Prospective cohort study—population based	Mothers experienced spontaneous preterm labour	Not exposed to tocolysis (atosiban and nifedipine)	Calcium channel blockers (atosiban and nifedipine)	Medical records	Neurodevelopmental disabilities included cerebral palsy; visual, hearing, or cognitive deficiencies; behavioural problems; or developmental coordination disorders	Standardized neurodevelopmental assessment scales	5.5 years

Abbreviations: ADHD, attention‐deficit/hyperactivity disorder; ASD, autism spectrum disorder.

### Quality assessment

Based on the NHLBI assessment tool, (Study Quality Assessment Tools) all the included studies achieved a good quality score, except for one RCT which was graded as fair quality (Tables 2 and 3). No additional eligible studies were identified from the reference lists of included studies.

**TABLE 2:** Quality assessment of observational studies.

Criteria	Fitton et al. (2021)	Hisle‐Gorman et al. (2018)	Jong et al. (2010)	Chan et al. (2010)	Huisjes et al. (1986)	Lorthe et al. (2024)	Plouchart et al. (2024)
1. Was the research question or objective in this paper clearly stated?	Yes	Yes	Yes	Yes	Yes	Yes	Yes
2. Was the study population clearly specified and defined?	Yes	Yes	Yes	Yes	Yes	Yes	Yes
3. Was the participation rate of eligible persons at least 50%?	Yes	NR	Yes	NR	NR	Yes	Yes
4. Were all the subjects selected or recruited from the same or similar populations (including the same time‐period)? Were inclusion and exclusion criteria for being in the study prespecified and applied uniformly to all participants?	Yes	Yes	Yes	Yes	Yes	Yes	Yes
5. Was a sample size justification, power description, or variance and effect estimates provided?	No	No	No	NR	NR	Yes	Yes
6. For the analyses in this paper, were the exposure(s) of interest measured prior to the outcome(s) being measured?	Yes	No	Yes	Yes	Yes	Yes	Yes
7. Was the timeframe sufficient so that one could reasonably expect to see an association between exposure and outcome if it existed?	Yes	Yes	Yes	Yes	Yes	Yes	Yes
8. For exposures that can vary in amount or level, did the study examine different levels of the exposure as related to the outcome (e.g., categories of exposure, or exposure measured as continuous variable)?	Yes	Yes	Yes	Yes	Yes	Yes	Yes
9. Were the exposure measures (independent variables) clearly defined, valid, reliable, and implemented consistently across all study participants?	Yes	Yes	Yes	Yes	Yes	Yes	Yes
10. Was the exposure(s) assessed more than once over time?	No	No	No	Yes	Yes	No	No
11. Were the outcome measures (dependent variables) clearly defined, valid, reliable, and implemented consistently across all study participants?	Yes	Yes	Yes	Yes	Yes	Yes	Yes
12. Were the outcome assessors blinded to the exposure status of participants?	No	No	No	Yes	No	No	No
13. Was loss to follow‐up after baseline 20% or less?	No	Yes	No	Yes	Yes	No	No
14. Were key potential confounding variables measured and adjusted statistically for their impact on the relationship between exposure(s) and outcome(s)?	Yes	Yes	Yes	Yes	NR	Yes	Yes
Total (out of 14)	10	9	10	12	10	12	11

Abbreviations: CD, cannot determine; NA, not applicable; NR, not reported.

**TABLE 3:** Quality assessment of controlled intervention studies.

Criteria	Cockburn et al. (1982)	van Vliet et al. (2015)	Bortolus et al. (2000)	Ounsted et al. (1980)
1. Was the study described as randomized, a randomized trial, a randomized clinical trial, or an RCT?	Yes	Yes	Yes	Yes
2. Was the method of randomization adequate (i.e., use of randomly generated assignment)?	Yes	Yes	Yes	Yes
3. Was the treatment allocation concealed (so that assignments could not be predicted)?	CD	Yes	Yes	No
4. Were study participants and providers blinded to treatment group assignment?	No	Yes	No	No
5. Were the people assessing the outcomes blinded to the participants' group assignments?	No	Yes	No	No
6. Were the groups similar at baseline on important characteristics that could affect outcomes (e.g., demographics, risk factors, co‐morbid conditions)?	No	Yes	Yes	Yes
7. Was the overall drop‐out rate from the study at endpoint 20% or lower of the number allocated to treatment?	Yes	No	No	Yes
8. Was the differential drop‐out rate (between treatment groups) at endpoint 15% points or lower?	Yes	NR	No	Yes
9. Was there high adherence to the intervention protocols for each treatment group?	Yes	Yes	No	Yes
10. Were other interventions avoided or similar in the groups (e.g., similar background treatments)?	Yes	Yes	Yes	Yes
11. Were outcomes assessed using valid and reliable measures, implemented consistently across all study participants?	Yes	Yes	Yes	Yes
12. Did the authors report that the sample size was sufficiently large to be able to detect a difference in the main outcome between groups with at least 80% power?	No	No	No	No
13. Were outcomes reported or subgroups analysed prespecified (i.e., identified before analyses were conducted)?	Yes	Yes	Yes	Yes
14. Were all randomized participants analysed in the group to which they were originally assigned, that is, did they use an intention‐to‐treat analysis?	No	Yes	No	Yes
Overall score (out of 14)	8	11	7	10

Abbreviations: CD, cannot determine; NA, not applicable; NR, not reported.

### Calcium channel blockers

Two RCTs of nifedipine (Bortolus et al., 2000; van Vliet et al., 2015), two prospective cohort studies (Lorthe et al., 2024; Plouchart et al., 2024), and one case‐control study (Hisle‐Gorman et al., 2018) investigated associations between prenatal exposure to calcium channel blockers and a range of neurodevelopmental outcomes (Table 1). An RCT of 170 children reported an association between prenatal nifedipine exposure and fine motor impairment at two years of age (Table 4) (van Vliet et al., 2015). However, no significant associations were observed with communication, gross motor movements, personal social problems, or developmental delay. Furthermore, the study suggested reduced risk of poor problem solving compared with children not exposed to nifedipine prenatally. Another RCT of 190 children reported no associations between prenatal nifedipine exposure and gross/fine motor movements, hearing, sight, or language at 18 months of age (Bortolus et al., 2000). In a case‐control study, 8760 children with ASD were more likely to have had mothers with hypertension who were on anti‐hypertensive treatment but did not establish a direct connection with antihypertensive medication use, including calcium channel blockers (Table 4) (Hisle‐Gorman et al., 2018).

**TABLE 4:** Results reported by eligible studies.

Authors/publication	Exposure	Outcome	Age when outcome measured	Results
1. Cockburn et al. (1982)	‘Specific antihypertensive treatment (mainly methyldopa) until delivery’	Copying	7 ½ years	59.81 ± 9.17^d^ versus 60.42 ± 9.47 (*t* = 0.46)^a^
		Verbal tactile matching		54.76 ± 8.54 versus 52.60 ± 8.22 (*t* = 1.77)
		Delayed visual recall		51.11 ± 12.71 versus 52.01 ± 12.72 (*t* = 0.49)
		Immediate visual recall		53.27 ± 10.55 versus 52.80 ± 10.98 (*t* = 0.30)
		Recall of designs		53.18 ± 9.99 versus 52.76 ± 8.88 (*t* = 0.31)
		Recall of digits		53.41 ± 10.26 versus 54.40 ± 11.58 (*t* = 0.62)
		Visual recognition		52.88 ± 10.70 versus 53.63 ± 11.84 (*t* = 0.46)
		Basic arithmetic		55.89 ± 9.66 versus 57.24 ± 9.89 (*t* = 0.96)
		Word definitions		51.53 ± 9.66 versus 50.83 ± 8.76 (*t* = 0.52)
		Verbal fluency		56.19 ± 7.51 versus 57.37 ± 6.77 (*t* = 1.14)
		Reading quotient		106.32 ± 20.52 versus 105.88 ± 18.79 (*t* = 0.15)
2. Bortolus et al. (2000)	Nifedipine	Gross/fine motor	18 months	4.3%^e^ versus 5.3%^f^
		Hearing/sight		3.2% versus 3.2%
		Language		9.6% versus 7.4%
3. Fitton et al. (2021)	≥1 antihypertensive	Gross motor	2.5 years	Hypertension untreated: OR 1.18 (99% CI 0.83–1.68)
				Exposure during pregnancy: OR 1.14 (99% CI 0.60–2.15)
		Fine motor		Hypertension untreated: OR 1.04 (99% CI 0.74–1.46)
				Exposure during pregnancy: OR 1.21 (99% CI 0.69–2.14)
		Vision		Hypertension untreated: OR 0.99 (99% CI 0.65–1.53)
				Exposure during pregnancy: OR 1.51 (99% CI 0.81–2.80)
		Hearing		Hypertension untreated: OR 0.96 (99% CI 0.64–1.44)
				Exposure during pregnancy: OR 0.91 (99% CI 0.43–1.93)
		Speech		Hypertension untreated: OR 1.04 (99% CI 0.93–1.17)
				**Exposure during pregnancy: OR 1.21 (99% CI 1.00–1.47)**
		Attention		Hypertension untreated: OR 1.13 (99% CI 0.92–1.40)
				Exposure during pregnancy: OR 1.00 (99% CI 0.65–1.52)
		Emotional		Hypertension untreated: OR 1.01 (99% CI 0.76–1.36)
				Exposure during pregnancy: OR 1.15 (99% CI 0.69–1.93)
		Behavioural		Hypertension untreated: OR 1.08 (99% CI 0.90–1.28)
				Exposure during pregnancy: OR 1.18 (99% CI 0.86–1.61)
		Social developmental problems		Hypertension untreated: OR 1.14 (99% CI 0.92–1.43)
				Exposure during pregnancy: OR 0.98 (99% CI 0.63–1.51)
4. Hisle‐Gorman et al. (2018)	Calcium channel blockers, β‐blockers	ASD	2–18 years	**Diagnosis only: OR 1.10 (95% CI 1.02–1.18)**
				**Diagnosis and prescription: OR 1.35 (95% CI 1.07–1.71)**
				Prescription only: OR 1.09 (95% CI 0.89–1.35)
5. Pasker‐De Jong et al. (2010)	Labetalol	ADHD	4–10 years	**OR 4.1 (95% CI 1.2–13.9)**
		Sleeping disorders		OR 1.4 (95% CI 0.2–10.2)
		IQ (TAKIT)		1.5^c^ (95% CI –4.1 to 7.1)
		Concentration score		1.5 (95% CI –7.7 to 10.7)
		Gross motor development		−0.1 (95% CI –2.0 to 1.8)
		Fine motor development		0.6 (95% CI –3.7 to 4.8)
		Visual memory		1.0 (95% CI –1.0 to 3.1)
		Associative memory		0.6 (95% CI –0.6 to 1.8)
		Auditory memory		−0.2 (95% CI –0.7 to 0.4)
	Methyldopa	Sleeping disorders		OR 4.5 (95% CI 0.9–23.2)
6. van Vliet et al. (2015)	Nifedipine	Fine motor scale	2 years	**OR 3.43 (95% CI 1.29‐9.14) (*p* = 0.01)**
		Poor problem‐solving		**OR 0.27 (95% CI 0.08‐0.95) (*p* = 0.04)**
		Communication scale		OR 0.56 (95% CI 0.25–1.26) (*p* = 0.16)
		Gross motor scale		OR 0.41 (95% CI 0.15–1.14) (*p* = 0.09)
		Personal social scale		OR 0.88 (95% CI 0.43–1.78) (*p* = 0.72)
		Developmental delay		OR 0.50 (95% CI 0.22–1.13) (*p* = 0.10)
7. Ounsted et al. (1980)	Methyldopa Specific antihypertensive treatment (mainly methyldopa) until delivery	Gross‐motor sector	4 years	Boys: 8.95 ± 2.76^d^ versus 8.56 ± 2.71, *F* = 0.32 (*p* > 0.05)^b^
				**Girls: 9.97 ± 2.68 versus 9.22 ± 2.86, *F* = 3.78 (*p* < 0.05)**
		Fine‐motor sector		**Boys: 8.55 ± 2.85 versus 8.11 ± 2.49, *F* = 3.20 (*p* < 0.05)**
				Girls: 10.21 ± 3.28 versus 9.29 ± 2.37, *F* = 1.20 (*p* > 0.05)
		Visuo‐motor sector		**Boys: 9.10 ± 2.93 versus 8.34 ± 2.84, *F* = 3.72 (*p* < 0.05)**
				Girls: 9.18 ± 3.51 versus 8.92 ± 3.25, *F* = 2.28 (*p* > 0.05)
		Language		Boys: 10.13 ± 2.97 versus 9.37 ± 3.02, *F* = 1.11 (*p* > 0.05)
				Girls: 10.17 ± 3.11 versus 9.795 ± 3.00, *F* = 0.57 (*p* > 0.05)
		Comprehension		Boys: 9.90 ± 2.92 versus 9.00 ± 2.93, *F* = 0.86 (*p* > 0.05)
				Girls: 9.56 ± 2.78 versus 9.33 ± 2.54, *F* = 0.66 (*p* > 0.05)
8. Huisjes et al. (1986)	Clonidine	Neurological examination	3–9 years	No
		School performance		No
		Behavioural characteristics		No
		Hyperactivity		Excess
		Sleep disturbances		**(*p* < 0.05)**
9. Chan et al. (2010)	Labetalol	Full‐scale IQ	3–7 years	Full‐scale IQ: 109.60 ± 8.20^d^ versus 111.90 ± 11.39 (*p* = 0.647)
		Performance		Performance IQ: 104.80 ± 8.69 versus 110.19 ± 12.91 (*p* = 0.186)
		Verbal		Verbal IQ: 112.27 ± 11.05 versus 11.21 ± 11.98 (*p* = 0.922)
	Methyldopa	Full‐scale IQ		**Full‐scale IQ: 105.24** ± **12.46 versus 111.90 ± 11.39 (*p* = 0.043)**
		Performance		**Performance IQ: 98.80** ± **16.16 versus 110.19** ± **12.91 (*p* = 0.002)**
		Verbal		Verbal IQ: 109.64 ± 11.01 versus 111.21 ± 11.98 (*p* = 0.85)
10. Lorthe et al. (2024)	Calcium channel blockers versus to tocolysis (atosiban and nifedipine)	Survival without moderate to severe neurodevelopmental disabilities	5.5 years	OR 0.92 (95% CI 0.52–1.62) (*p* = 0.62)
		Survival without any neurodevelopmental disabilities		OR 1.02 (95% CI 0.65–1.61) (*p* = 0.62)
11. Plouchart et al. (2024)	Calcium channel blockers versus to tocolysis (atosiban and nifedipine)	Neurodevelopmental disabilities	5.5 years	RR 1.11 (95% CI 0.85–1.45) (*p* = 0.44)

*Note*: Bold = statistically significant.

Abbreviations: ADHD, attention‐deficit/hyperactivity disorder; ASD, autism spectrum disorder; CI, confidence interval; OR, odds ratio; RR, relative risk.

^a^ These are standardised scores with a mean value of 50 and a standard deviation of 10.

^b^ Each sector had been standardised to give a mean score of 10 and an SD of 3.

^c^ Mean difference.

^d^ Mean ± SD.

^e^ No. of treated group.

^f^ No. of untreated group.

The two prospective cohort studies (Lorthe et al., 2024; Plouchart et al., 2024) investigated the impact of calcium channel blockers, including nifedipine, used as tocolytics for preterm labour, on neurodevelopmental outcomes of offspring. A population‐based cohort study of 712 singleton pregnancies using EPIPAGE‐2 data found no association between exposure to tocolysis and any neurodevelopmental disabilities by 5.5 years of age. Another prospective cohort study included 1055 mothers and followed up 1320 children born to these mothers until the age of 5.5 years. This study also reported no difference in risk of neurodevelopmental disabilities between children exposed to tocolysis and those who were not.

### Beta‐blockers and alpha agonists

In a cohort study of 202 children, prenatal exposure to labetalol was associated with ADHD at 4–10 years of age (odds ratio [OR] 4.1, 95% confidence interval [CI]: 1.2–13.9) but the wide confidence intervals reflect the lack of precision in the estimate of effect size. However, there were no associations with sleep disorders, intelligence quotient (IQ), memory, concentration, and gross or fine motor skills (Table 4) (Pasker‐De Jong et al., 2010). In a retrospective case‐control study of 44 children, prenatal exposure to clonidine was associated with hyperactivity and sleep disturbance at between 3 and 9 years of age (Huisjes et al., 1986). However, there was no association with behaviour, school performance or findings on neurological examination. As reported above, a case‐control study reported an association between ASD and prenatal use of either calcium channel blockers or beta‐blockers by mothers with recorded hypertension (Hisle‐Gorman et al., 2018). Finally, a cohort study of 99 children found no association between prenatal exposure to labetalol and either performance or verbal IQ at between 2 and 7 years of age (Chan et al., 2010).

### Methyldopa exposure

In a cohort study of 99 children, prenatal exposure to methyldopa was associated with a significantly lower overall IQ score (105.24 ± 12.46 compared to 111.90 ± 11.39, *p* = 0.043) at between 3 and 7 years of age (Chan et al., 2010). This result was primarily attributed to performance rather than verbal intelligence (Table 4). In an RCT that randomised 168 hypertensive pregnant women to methyldopa or no treatment (Table 1) (Ounsted et al., 1980), there was no significant difference between any developmental sector score in either boys or girls. Male offspring had significantly better fine and visuo‐motor skills at 4 years of age (Table 4) whilst female offspring had better gross motor skills. However, the mean scores for all sectors were consistently higher in the treated group than untreated ones in both genders (Table 4). Similarly, in an RCT in which 195 pregnant women were randomised to an unspecified antihypertensive (mainly methyldopa), there were no significant differences in a wide range of offspring outcomes at 7.5 years of age: copying, verbal tactile matching and fluency, immediate and delayed visual recall, digits recall, visual recognition, basic arithmetic, word definitions and reading quotient (Table 4) (Cockburn et al., 1982). Children in both groups had higher ability scores than the standardisation sample. In a cohort study of 202 children, prenatal exposure to methyldopa was not significantly associated with sleep disorders compared to bed‐rest groups (OR 4.5, 95% CI 0.9–23.2) at between 4 and 10 years of age (Pasker‐De Jong et al., 2010).

### Unspecified antihypertensive drugs

In a retrospective cohort study of 117, 963 singleton pregnancies, untreated hypertension during pregnancy was not associated with adverse outcomes at 2.5 years of age, whereas treatment with one or more antihypertensive medications was associated with an increased risk of speech impairment but not gross/fine motor, hearing or vision impairments (Fitton et al., 2021).

## DISCUSSION

This review found that 6 of the 11 eligible studies identified at least one adverse neurodevelopmental outcome associated with prenatal antihypertensive exposure. Different antihypertensive medication classes (beta‐blockers, calcium channel blockers, methyldopa, alpha agonists) were investigated, and results across studies were inconsistent. Associations were observed by at least one study in relation to ASD, ADHD, and impairments in sleep, speech, motor skills, or IQ. However, where multiple studies examined the same medication class, the findings were often contradictory (Table 5).

**TABLE 5:** Summary of the examined antihypertensive medications and neurodevelopmental and educational outcomes.

			Neurodevelopmental outcomes																							
Authors/Publication	Exposure	Size	CP	CD	ADHD	ASD	Sleeping disorders	Speech problems	Gross motor problem	Fine motor problem	Visuo‐motor problem	Hearing problem	Language issue	Emotional and behavioural difficulties	Development problem	NDD	Personal social and communication scale	Lower IQ scores	Visual‐associative—auditory memory	Concentration score	Neurological examination	School performance	Hyperactivity	Comprehension	Abilities skills difficulties	Better problem‐solving
Cockburn et al. (1982)	Methyldopa	195	−	−	−	−	−	−	−	−	−	−	−	−	−	−	−	−	−	−	−	−	−	−	+	−
Bortolus et al. (2000)	Nifedipine	190	−	−	−	−	−	−	+	+	−	−	+	−	−	−	−	−	−	−	−	−	−	−	−	−
Fitton et al. (2021)	≥1 antihypertensive	117,963	−	−	−	−	−	+	+	+	+	+	+	+	+	−	−	−	−	−	−	−	−	−	−	−
Hisle‐Gorman et al. (2018)	Calcium channel blockers	8760	−	−	−	+	−	−	−	−	−	−	−	−	−	−	−	−	−	−	−	−	−	−	−	−
	Beta‐blockers		−	−	−	+	−	−	−	−	−	−	−	−	−	−	−	−	−	−	−	−	−	−	−	−
Pasker‐De Jong et al. (2010)	Labetalol	202	−	−	+	−	−	−	−	−	−	−	−	−	−	−	−	−	+	+	−	−	−	−	−	−
	Methyldopa		−	−	−	−	+	−	−	−	−	−	−	−	−	−	−	−	−	−	−	−	−	−	−	−
Van Vliet et al. (2015)	Nifedipine	170	−	−	−	−	−	−	+	+	−	−	−	−	+	−	+	−	−	−	−	−	−	−	−	+
Ounsted et al. (1980)	Methyldopa	168	−	−	−	−	−	−	+	+	+	−	+	−	−	−	−	−	−	−	−	−	−	+	−	−
Huisjes et al. (1986)	Clonidine	44	−	−	−	−	+	−	−	−	−	−	−	+	−	−	−	−	−	−	+	+	+	−	−	−
Chan et al. (2010)	Labetalol	99	−	−	−	−	−	−	−	−	−	−	−	−	−	−	−	+	−	−	−	−	−	−	−	−
	Methyldopa		−	−	−	−	−	−	−	−	−	−	−	−	−	−	−	+	−	−	−	−	−	−	−	−
Lorthe et al. (2024)	Calcium channel blockers	712	+	−	−	−	−	−	−	−	−	−	−	+	+	+	−	+	−	−	−	−	−	−	−	−
Plouchart et al. (2024)	Calcium channel blockers	1320	+	+	−	−	−	−	−	+	−	+	−	+	+	+	−	−	−	−	−	−	−	−	−	−

*Note:* Abilities skills difficulties: copying, verbal tactile matching, delayed visual recall, immediate visual recall, recall of designs, recall of digits, visual recognition, basic arithmetic, word definitions, verbal fluency, reading quotient. (+): examined; (−): not examined; (+) in red colour: examined and significant result.

Abbreviations: ADHD, attention‐deficit/hyperactivity disorder; ASD, autism spectrum disorder; CD, cognitive deficiencies; CP, cerebral palsy; IQ: intelligence quotient; NDD, neurodevelopmental disabilities.

This study was strengthened by a wide variety of reported outcomes including neurodevelopmental problems or developmental delay; growth retardation; IQ, problem solving or cognitive problems; speech and hearing loss, gross and fine motor problems; problems sleeping; ADHD and ASD. Ten studies investigated different individual medications, classes of medication, or combinations of these, and one investigated exposure to one or more unspecified anti‐hypertensive medications. The quality of eligible studies was assessed and reported. However, large heterogeneity between the studies precluded conducting a meta‐analysis. This heterogeneity stemmed from differences in study design, outcome measures, medication classes, and dosages, as well as the variability in treatment duration and definitions of exposure. Of the four classes of antihypertensives, only beta‐blockers, methyldopa and calcium channel blockers were investigated by at least two studies each.

Inconsistencies in findings may, in part, be attributed to these methodological differences, as well as varying population characteristics and healthcare settings. This can be in relation to the differences in exclusion criteria of the eligible studies, such as propranolol being excluded from some studies (Fitton et al., 2021) but not others, and studies either excluding (Chan et al., 2010) or including (Fitton et al., 2021) women taking more than one antihypertensive medication. Inconsistencies may also be due to differences in the timing of exposure analysed (first, second, or third trimester exposure or exposure throughout pregnancy) or failure to stratify results by exposure period.

For example, one RCT reported no association between nifedipine taken between 12 and 34 weeks of gestation and neurodevelopmental outcomes (Bortolus et al., 2000), whilst another study observed reduced fine motor function associated when nifedipine use started between 26 and 32 weeks of gestation (van Vliet et al., 2015). Two cohort studies (Lorthe et al., 2024; Plouchart et al., 2024) reported no increased risk associated with tocolysis administered during 24–31 weeks, and 24–34 weeks, respectively. These differences might be due to variation in follow up period, time of exposure, study design, sample size and population. The latter two high‐quality large cohort studies suggested that receipt of antenatal tocolysis, including nifedipine, after preterm prelabour rupture of membranes, did not show any significant impact on the risk of long‐term neurodevelopmental disabilities outcomes. However, the null findings may be explained by both studies having excluded pregnant women with a recorded diagnosis of HDP. Evidence has found that hypertensive conditions may exert a stronger influence on the intended outcomes than medication treatment alone. Hisle‐Gorman et al. (2018) revealed that ASD was associated with hypertensive conditions or their antihypertensive treatments (i.e., calcium channel blockers and beta‐blockers) but not with treatment alone.

It is difficult to draw definitive recommendations from the existing literature due to the heterogeneity in study design, definition and measurement of both exposure and outcomes, and ability to differentiate between the treatment and the underlying condition. Therefore, the primary recommendation is for future studies to address these limitations; either through large scale studies that provide definitive evidence or via multiple smaller scale studies that adopt consistent approaches to study design, definitions and measurements.

Study samples were generally small, limiting the statistical power of studies and the precision of their estimated effect sizes. Of the 11 articles, 7 studied between 44 and 202 children. Furthermore, exclusion of non‐English studies and grey publication may have resulted in the exclusion of otherwise eligible studies. Studies varied in the extent to which they controlled for potential confounding and the predominantly short‐term follow‐up of the studies limited insights into long‐term effects. Whilst Hemolysis, Elevated Liver Enzymes, Low Platelet Count syndrome (a specific type of preeclampsia) was not included as a search term, a subsequent search of the term did not identify any additional eligible studies.

While some studies adjusted for maternal age, socioeconomic status, and parity, very few adjusted for lifestyle factors such as smoking or alcohol use. It is essential to adjust for potential confounders that can bias true associations. For example, smoking is more prevalent among individuals with hypertension conditions (Bowman et al., 2007; Jareebi, 2024) and maternal smoking is also associated with neurodevelopmental outcomes (Ediger et al., 2019).

A critical question regarding offspring outcomes is how to disentangle the effects of maternal hypertension from the effect of antihypertensive medication. However, as highlighted by our review, not all the included studies considered the effect of both the underlying condition and its medication. Similarly, the conditions that comprise hypertensive disorders of pregnancy—chronic hypertension, gestational hypertension and pre‐eclampsia—are associated with different levels of risk for both mother and child. However, many studies did not differentiate between these conditions in their analyses. Future research must account for the interplay between maternal disease and medication exposure by comparing treated and untreated disease groups. This would allow for a more nuanced understanding of the relative contributions of these factors to neurodevelopmental and educational outcomes in children.

## CONCLUSION

The evidence from current literature surrounding the impact of prenatal exposure to antihypertensive medication on neurodevelopmental outcomes remains inconclusive, hindered by small, heterogeneous studies and small sample sizes. Future research should focus on larger‐scale, comprehensive studies with standardized methodologies to better understand these associations.

## AUTHOR CONTRIBUTIONS

**Shrifah Alkhalaf**: Data curation; formal analysis; funding acquisition; investigation; methodology; software; validation; visualization; writing—original draft; writing—review and editing. **Sarjit Singh**: Data curation; investigation; methodology; software; validation; visualization; writing—review and editing. **Jill P. Pell**: Investigation; methodology; supervision; validation; visualization; writing—review and editing. **Scott M. Nelson**: Investigation; methodology; validation; visualization; writing—review and editing. **Daniel F. Mackay**: Investigation; methodology; supervision; validation; visualization; writing—review and editing. **Michael Fleming**: Conceptualization; funding acquisition; investigation; methodology; project administration; resources; supervision; validation; visualization; writing—review and editing. All authors agreed the study design, interpreted the results, contributed revisions, reviewed and approved the final version of the manuscript, and agree to be accountable for all aspects of the work. Michael Fleming and Shrifah Alkhalaf are guarantors for the study.

## CONFLICT OF INTEREST STATEMENT

The authors declare no conflicts of interest.

## ETHICAL CONSIDERATIONS

Not required. Secondary analysis of published data. No primary data collection.

## Supporting information

Supporting Information S1

## Data Availability

Data sharing not applicable to this article as no datasets were generated or analysed during the current study.
