# Supplementary material for: Prenatal exposure to antihypertensive medication: A systematic review of neurodevelopmental and educational outcomes
Source: JCPP Adv. 2025 Dec 19;6(3):e70080. doi: 10.1002/jcv2.70080 (PMC13338986; doi:10.1002/jcv2.70080)
Supplement: Supplementary file 1 [file JCV2-6-e70080-s001.docx]

**Appendix**

**Ovid MEDLINE(R) ALL <1946 to July 04, 2025>**

1	Pregnancy/

2	Pre-Eclampsia/

3	Eclampsia/

4	Toxemia/

5	Hypertension, Pregnancy-Induced/

6	Pregnancy Complications, Cardiovascular/

7	(hypertens* and pregnan*).mp.

8	(hypertens* adj pregnan*).mp.

9	(pre-eclamp* or preeclamp* or eclamp* or toxemia or toxaemia or Maternal hypertens*).mp.

10	(gestational adj hypertens*).mp.

11	(gestational and hypertens*).mp.

12	(pregnan* or prenatal or perinatal or maternal).mp.

13	1 or 2 or 3 or 4 or 5 or 6 or 7 or 8 or 9 or 10 or 11 or 12

14	Antihypertensive Agents/

15	Thiazides/

16	Sodium Chloride Symporter Inhibitors/

17	Sodium Potassium Chloride Symporter Inhibitors/

18	(amiloride or benzothiadiazine or bendroflumethiazide or bumetanide or chlorothiazide or cyclopenthiazide or furosemide or hydrochlorothiazide or hydroflumethiazide or methyclothiazide or metolazone or polythiazide or trichlormethiazide or veratide or thiazide*).mp.

19	(chlorthalidone or chlortalidone or phthalamudine or chlorphthalidolone or oxodoline or thalitone or hygroton or indapamide or metindamide).mp.

20	(Dihydralazine or Acetazolamide or sodium nitroprusside or clonidine hydrochloride or Potassium-sparing agents or loop diuretic or sodium nitroprusside or moxonidine or renin inhibitor).mp.

21	15 or 16 or 17 or 18 or 19 or 20

22	Angiotensin-Converting Enzyme Inhibitors/

23	angiotensin converting enzyme inhibit*.mp.

24	(alacepril or altiopril or ancovenin or benazepril or captopril or ceranapril or ceronapril or cilazapril or deacetylalacepril or delapril or derapril or enalapril or epicaptopril or fasidotril or fosinopril or foroxymithine or gemopatrilat or idapril or imidapril or indolapril or libenzapril or lisinopril or moexipril or moveltipril or omapatrilat or pentopril* or perindopril* or pivopril or quinapril* or ramipril* or rentiapril or saralasin or s nitrosocaptopril or spirapril* or temocapril* or teprotide or trandolapril* or utibapril* or zabicipril* or zofenopril* or Aceon or Accupril or Altace or Capoten or Lotensin or Mavik or Monopril or Prinivil or Univas or Vasotec or Zestril).mp.

25	22 or 23 or 24

26	Angiotensin Receptor Antagonists/

27	(abitesartan or azilsartan or candesartan or elisartan or embusartan or eprosartan or forasartan or irbesartan or losartan or milfasartan or olmesartan or saprisartan or tasosartan or telmisartan or valsartan or zolasartan or Atacand or Avapro or Benicar or Cozaar or Diovan or Micardis or Teveten).mp.

28	26 or 27

29	Calcium Channel Blockers/

30	(amlodipine or aranidipine or barnidipine or bencyclane or benidipine or bepridil or cilnidipine or cinnarizine or clentiazem or darodipine or diltiazem or efonidipine or elgodipine or etafenone or fantofarone or felodipine or fendiline or flunarizine or gallopamil or isradipine or lacidipine or lercanidipine or lidoflazine or lomerizine or manidipine or mibefradil or nicardipine or nifedipine or niguldipine or nilvadipine or nimodipine or nisoldipine or nitrendipine or perhexiline or prenylamine or semotiadil or terodiline or tiapamil or verapamil or Cardizem CD or Dilacor XR or Tiazac or Cardizem Calan or Isoptin or Calan SR or Isoptin SR Coer or Covera HS or Verelan PM).mp.

31	(Nondihydropyridines or Dihydropyridines or ACE inhibitors or ACEi or Angiotensin II receptor blockers or angiotensin II receptor).mp.

32	29 or 30 or 31

33	(methyldopa or alphamethyldopa or amodopa or dopamet or dopegyt or dopegit or dopegite or emdopa or hyperpax or hyperpaxa or methylpropionic acid or dopergit or meldopa or methyldopate or medopa or medomet or sembrina or aldomet or aldometil or aldomin or hydopa or methyldihydroxyphenylalanine or methyl dopa or mulfasin or presinol or presolisin or sedometil or sembrina or taquinil or dihydroxyphenylalanine or methylphenylalanine or methylalanine or alpha methyl dopa).mp.

34	(reserpine or serpentina or rauwolfia or serpasil).mp.

35	(clonidine or adesipress or arkamin or caprysin or catapres* or catasan or chlofazolin or chlophazolin or clinidine or clofelin* or clofenil or clomidine or clondine or clonistada or clonnirit or clophelin* or dichlorophenylaminoimidazoline or dixarit or duraclon or gemiton or haemiton or hemiton or imidazoline or isoglaucon or klofelin or klofenil or normopresan or paracefan or tesno timelets).mp.

36	Hydralazine/

37	(hydralazin* or hydrallazin* or hydralizine or hydrazinophtalazine or hydrazinophthalazine or hydrazinophtalizine or dralzine or hydralacin or hydrolazine or hypophthalin or hypoftalin or hydrazinophthalazine or idralazina or 1-hydrazinophthalazine or apressin or nepresol or apressoline or apresoline or apresolin or alphapress or alazine or idralazina or lopress or plethorit or praeparat).mp.

38	33 or 34 or 35 or 36 or 37

39	Adrenergic beta-Antagonists/

40	(acebutolol or adimolol or afurolol or alprenolol or amosulalol or arotinolol or atenolol or befunolol or betaxolol or bevantolol or bisoprolol or bopindolol or bornaprolol or brefonalol or bucindolol or bucumolol or bufetolol or bufuralol or bunitrolol or bunolol or bupranolol or butofilolol or butoxamine or carazolol or carteolol or carvedilol or celiprolol or cetamolol or chlortalidone cloranolol or cyanoiodopindolol or cyanopindolol or deacetylmetipranolol or diacetolol or dihydroalprenolol or dilevalol or epanolol or esmolol or exaprolol or falintolol or flestolol or flusoxolol or hydroxybenzylpinodolol or hydroxycarteolol or hydroxymetoprolol or indenolol or iodocyanopindolol or iodopindolol or iprocrolol or isoxaprolol or labetalol or landiolol or levobunolol or levomoprolol or medroxalol or mepindolol or methylthiopropranolol or metipranolol or metoprolol or moprolol or nadolol or oxprenolol or penbutolol or pindolol or nadolol or nebivolol or nifenalol or nipradilol or oxprenolol or pafenolol or pamatolol or penbutolol or pindolol or practolol or primidolol or prizidilol or procinolol or pronetalol or propranolol or proxodolol or ridazolol or salcardolol or soquinolol or sotalol or spirendolol or talinolol or tertatolol or tienoxolol or tilisolol or timolol or tolamolol or toliprolol or tribendilol or xibenolol).mp.

41	(beta-adrenoceptor blocking drugs or vasodilator antihypertensive drugs or centrally acting antihypertensive drugs or Adrenergic alpha-Antagonists or adrenergic receptor antagonist or adrenergic receptor blocker or alpha-2 adrenergic receptor agonist).mp.

42	Adrenergic alpha-Antagonists/

43	(alfuzosin or bunazosin or doxazosin or metazosin or neldazosin or prazosin or silodosin or tamsulosin or terazosin or tiodazosin or trimazosin or urapidil).mp.

44	39 or 40 or 41 or 42 or 43

45	14 or 21 or 25 or 28 or 32 or 38 or 44

46	Neurodevelopmental Disorders/

47	Cognition Disorders/

48	Neurocognitive Disorders/

49	Autism Spectrum Disorder/

50	Attention Deficit Disorder with Hyperactivity/

51	Motor Disorders/

52	Epilepsy/

53	Intellectual Disability/

54	(longer term outcome or long term outcome* or long-term outcome*).mp.

55	Child Development/

56	Child development*.mp.

57	Executive Function/

58	Executive function*.mp.

59	Cognitive Dysfunction/

60	Neuropsychology/

61	neuropsycholog*.mp.

62	(neurodevelopment* or neurodevelopmental disorder* or educational difficult* or mental deficienc* or cognitive deficit* or poor performance or IQ or intelligence quotient or behavio* or behavioural disturbance* or social* or emotion* or motor impairment* or communication* or learning disabilit* or learning difficult* or learning disorder* or autism or intellectual disabilit* or intellectual impairment* or mental retardation* or attention deficit hyperactivity disorder* or attention deficit disorder* or ADHD or epilepsy or cerebral palsy or neurocognitive disorder* or cognitive impairment* or cognitive disabilit* or developmental disorder* or developmental disabilit* or development disabilit* or cognitive disturbance* or hypertensive pregnancy disorder* or hypertensive disorders of pregnancy or autism spectrum or autistic or autism spectrum disorder* or Asperger or Asperger's or Asperger's Syndrome or hyperactive* or overactive* or inattention or hyperkinetic disorder* or hyperkinet* or neurodevelopment or communication disorder* or motor disorder* or school performance).mp.

63	Congenital Abnormalities/

64	(congenital adj defect*).mp.

65	(congenital adj malformation*).mp.

66	(congenital adj anomal*).mp.

67	46 or 47 or 48 or 49 or 50 or 51 or 52 or 53 or 54 or 55 or 56 or 57 or 58 or 59 or 60 or 61 or 62 or 63 or 64 or 65 or 66

68	exp Child/

69	exp Infant/

70	(Baby or babies or offspring* or infant* or toddler* or preschool* or child* or kindergarten* or young person or youth or fetal or foetal or fetus or foetus or prenatal or pre school or pre-school).mp.

71	68 or 69 or 70

72	13 and 45 and 67 and 71

73	limit 72 to (english language and humans)

**Embase Ovid 1947-Present, updated daily 04/07/2025**

1	pregnancy/

2	preeclampsia/

3	eclampsia/

4	toxemia/

5	maternal hypertension/

6	Hypertension, Pregnancy-Induced.mp.

7	Pregnancy Complications, Cardiovascular.mp.

8	(hypertens* and pregnan*).mp.

9	(hypertens* adj pregnan*).mp.

10	(pre-eclamp* or preeclamp* or eclamp* or toxemia or toxaemia or Maternal hypertens*).mp.

11	(gestational adj hypertens*).mp.

12	(gestational and hypertens*).mp.

13	(pregnan* or prenatal or perinatal or maternal).mp.

14	1 or 2 or 3 or 4 or 5 or 6 or 7 or 8 or 9 or 10 or 11 or 12 or 13

15	antihypertensive agent/

16	thiazide diuretic agent/

17	Sodium Chloride Symporter Inhibitors.mp.

18	Sodium Potassium Chloride Symporter Inhibitors.mp.

19	(amiloride or benzothiadiazine or bendroflumethiazide or bumetanide or chlorothiazide or cyclopenthiazide or furosemide or hydrochlorothiazide or hydroflumethiazide or methyclothiazide or metolazone or polythiazide or trichlormethiazide or veratide or thiazide*).mp.

20	(chlorthalidone or chlortalidone or phthalamudine or chlorphthalidolone or oxodoline or thalitone or hygroton or indapamide or metindamide).mp.

21	(Dihydralazine or Acetazolamide or sodium nitroprusside or clonidine hydrochloride or Potassium-sparing agents or loop diuretic or sodium nitroprusside or moxonidine or renin inhibitor).mp.

22	16 or 17 or 18 or 19 or 20 or 21

23	angiotensin converting enzyme inhibit*.mp.

24	(alacepril or altiopril or ancovenin or benazepril or captopril or ceranapril or ceronapril or cilazapril or deacetylalacepril or delapril or derapril or enalapril or epicaptopril or fasidotril or fosinopril or foroxymithine or gemopatrilat or idapril or imidapril or indolapril or libenzapril or lisinopril or moexipril or moveltipril or omapatrilat or pentopril* or perindopril* or pivopril or quinapril* or ramipril* or rentiapril or saralasin or s nitrosocaptopril or spirapril* or temocapril* or teprotide or trandolapril* or utibapril* or zabicipril* or zofenopril* or Aceon or Accupril or Altace or Capoten or Lotensin or Mavik or Monopril or Prinivil or Univas or Vasotec or Zestril).mp.

25	23 or 24

26	angiotensin receptor antagonist/

27	(abitesartan or azilsartan or candesartan or elisartan or embusartan or eprosartan or forasartan or irbesartan or losartan or milfasartan or olmesartan or saprisartan or tasosartan or telmisartan or valsartan or zolasartan or Atacand or Avapro or Benicar or Cozaar or Diovan or Micardis or Teveten).mp.

28	26 or 27

29	calcium channel blocking agent/

30	(amlodipine or aranidipine or barnidipine or bencyclane or benidipine or bepridil or cilnidipine or cinnarizine or clentiazem or darodipine or diltiazem or efonidipine or elgodipine or etafenone or fantofarone or felodipine or fendiline or flunarizine or gallopamil or isradipine or lacidipine or lercanidipine or lidoflazine or lomerizine or manidipine or mibefradil or nicardipine or nifedipine or niguldipine or nilvadipine or nimodipine or nisoldipine or nitrendipine or perhexiline or prenylamine or semotiadil or terodiline or tiapamil or verapamil or Cardizem CD or Dilacor XR or Tiazac or Cardizem Calan or Isoptin or Calan SR or Isoptin SR Coer or Covera HS or Verelan PM).mp.

31	(Nondihydropyridines or Dihydropyridines or ACE inhibitors or ACEi or Angiotensin II receptor blockers or angiotensin II receptor).mp.

32	29 or 30 or 31

33	(methyldopa or alphamethyldopa or amodopa or dopamet or dopegyt or dopegit or dopegite or emdopa or hyperpax or hyperpaxa or methylpropionic acid or dopergit or meldopa or methyldopate or medopa or medomet or sembrina or aldomet or aldometil or aldomin or hydopa or methyldihydroxyphenylalanine or methyl dopa or mulfasin or presinol or presolisin or sedometil or sembrina or taquinil or dihydroxyphenylalanine or methylphenylalanine or methylalanine or alpha methyl dopa).mp.

34	(reserpine or serpentina or rauwolfia or serpasil).mp.

35	(clonidine or adesipress or arkamin or caprysin or catapres* or catasan or chlofazolin or chlophazolin or clinidine or clofelin* or clofenil or clomidine or clondine or clonistada or clonnirit or clophelin* or dichlorophenylaminoimidazoline or dixarit or duraclon or gemiton or haemiton or hemiton or imidazoline or isoglaucon or klofelin or klofenil or normopresan or paracefan or tesno timelets).mp.

36	hydralazine/

37	(hydralazin* or hydrallazin* or hydralizine or hydrazinophtalazine or hydrazinophthalazine or hydrazinophtalizine or dralzine or hydralacin or hydrolazine or hypophthalin or hypoftalin or hydrazinophthalazine or idralazina or 1-hydrazinophthalazine or apressin or nepresol or apressoline or apresoline or apresolin or alphapress or alazine or idralazina or lopress or plethorit or praeparat).mp.

38	33 or 34 or 35 or 36 or 37

39	beta adrenergic receptor blocking agent/

40	(acebutolol or adimolol or afurolol or alprenolol or amosulalol or arotinolol or atenolol or befunolol or betaxolol or bevantolol or bisoprolol or bopindolol or bornaprolol or brefonalol or bucindolol or bucumolol or bufetolol or bufuralol or bunitrolol or bunolol or bupranolol or butofilolol or butoxamine or carazolol or carteolol or carvedilol or celiprolol or cetamolol or chlortalidone cloranolol or cyanoiodopindolol or cyanopindolol or deacetylmetipranolol or diacetolol or dihydroalprenolol or dilevalol or epanolol or esmolol or exaprolol or falintolol or flestolol or flusoxolol or hydroxybenzylpinodolol or hydroxycarteolol or hydroxymetoprolol or indenolol or iodocyanopindolol or iodopindolol or iprocrolol or isoxaprolol or labetalol or landiolol or levobunolol or levomoprolol or medroxalol or mepindolol or methylthiopropranolol or metipranolol or metoprolol or moprolol or nadolol or oxprenolol or penbutolol or pindolol or nadolol or nebivolol or nifenalol or nipradilol or oxprenolol or pafenolol or pamatolol or penbutolol or pindolol or practolol or primidolol or prizidilol or procinolol or pronetalol or propranolol or proxodolol or ridazolol or salcardolol or soquinolol or sotalol or spirendolol or talinolol or tertatolol or tienoxolol or tilisolol or timolol or tolamolol or toliprolol or tribendilol or xibenolol).mp.

41	(beta-adrenoceptor blocking drugs or vasodilator antihypertensive drugs or centrally acting antihypertensive drugs or Adrenergic alpha-Antagonists or adrenergic receptor antagonist or adrenergic receptor blocker or alpha-2 adrenergic receptor agonist).mp.

42	alpha adrenergic receptor blocking agent/

43	(alfuzosin or bunazosin or doxazosin or metazosin or neldazosin or prazosin or silodosin or tamsulosin or terazosin or tiodazosin or trimazosin or urapidil).mp.

44	39 or 40 or 41 or 42 or 43

45	15 or 22 or 25 or 28 or 32 or 38 or 44

46	mental disease/

47	Neurodevelopmental Disorders.mp.

48	cognitive defect/

49	Neurocognitive Disorders.mp.

50	autism/

51	attention deficit hyperactivity disorder/

52	motor dysfunction/

53	epilepsy/

54	intellectual impairment/

55	(longer term outcome or long term outcome* or long-term outcome*).mp.

56	child development/

57	Child development*.mp.

58	executive function/

59	Executive function*.mp.

60	neuropsychology/

61	neuropsycholog*.mp.

62	(neurodevelopment* or neurodevelopmental disorder* or educational difficult* or mental deficienc* or cognitive deficit* or poor performance or IQ or intelligence quotient or behavio* or behavioural disturbance* or social* or emotion* or motor impairment* or communication* or learning disabilit* or learning difficult* or learning disorder* or autism or intellectual disabilit* or intellectual impairment* or mental retardation* or attention deficit hyperactivity disorder* or attention deficit disorder* or ADHD or epilepsy or cerebral palsy or neurocognitive disorder* or cognitive impairment* or cognitive disabilit* or developmental disorder* or developmental disabilit* or development disabilit* or cognitive disturbance* or hypertensive pregnancy disorder* or hypertensive disorders of pregnancy or autism spectrum or autistic or autism spectrum disorder* or Asperger or Asperger's or Asperger's Syndrome or hyperactive* or overactive* or inattention or hyperkinetic disorder* or hyperkinet* or neurodevelopment or communication disorder* or motor disorder* or school performance).mp.

63	congenital disorder/

64	(congenital adj defect*).mp.

65	(congenital adj malformation*).mp.

66	(congenital adj anomal*).mp.

67	46 or 47 or 48 or 49 or 50 or 51 or 52 or 53 or 54 or 55 or 56 or 57 or 58 or 59 or 60 or 61 or 62 or 63 or 64 or 65 or 66

68	exp child/

69	exp infant/

70	(Baby or babies or offspring* or infant* or toddler* or preschool* or child* or kindergarten* or young person or youth or fetal or foetal or fetus or foetus or prenatal or pre school or pre-school).mp.

71	68 or 69 or 70

72	14 and 45 and 67 and 71

73	limit 72 to (human and english language)

**Database (3): PubMed - 04/07/2025**

(((((pregnancy[MeSH:noexp] OR "Hypertension, Pregnancy-Induced"[MeSH:noexp] OR "Pregnancy Complications, Cardiovascular"[MeSH:noexp] OR Pre-Eclampsia[MeSH:noexp] OR Eclampsia[MeSH:noexp] OR Toxemia[MeSH:noexp] OR "gestational hyperten*"[Title/Abstract] OR pre-eclamp*[Title/Abstract] OR preeclamp*[Title/Abstract] OR eclamp*[Title/Abstract] OR toxemia[Title/Abstract] OR toxaemia[Title/Abstract] OR "maternal hyperten*"[Title/Abstract] OR pregnan*[Title/Abstract] OR prenatal[Title/Abstract] OR perinatal[Title/Abstract] OR maternal[Title/Abstract])) AND ((humans[Filter]) AND (english[Filter]))) OR (((hypertens* AND pregnan*)) AND ((humans[Filter]) AND (english[Filter]))) OR (((gestational AND hypertens*)) AND ((humans[Filter]) AND (english[Filter])))) AND ((humans[Filter]) AND (english[Filter]))) AND ((("Antihypertensive Agents"[MeSH:noexp] OR Thiazides[MeSH:noexp] OR "Sodium Chloride Symporter Inhibitors"[MeSH:noexp] OR "Sodium Potassium Chloride Symporter Inhibitors"[MeSH:noexp] OR amiloride[Title/Abstract] OR benzothiadiazine[Title/Abstract] OR bendroflumethiazide[Title/Abstract] OR bumetanide[Title/Abstract] OR chlorothiazide[Title/Abstract] OR cyclopenthiazide[Title/Abstract] OR furosemide[Title/Abstract] OR hydrochlorothiazide[Title/Abstract] OR hydroflumethiazide[Title/Abstract] OR methyclothiazide[Title/Abstract] OR metolazone[Title/Abstract] OR polythiazide[Title/Abstract] OR trichlormethiazide[Title/Abstract] OR veratide[Title/Abstract] OR thiazide*[Title/Abstract] OR chlorthalidone[Title/Abstract] OR chlortalidone[Title/Abstract] OR phthalamudine[Title/Abstract] OR chlorphthalidolone[Title/Abstract] OR oxodoline[Title/Abstract] OR thalitone[Title/Abstract] OR hygroton[Title/Abstract] OR indapamide[Title/Abstract] OR metindamide[Title/Abstract] OR Dihydralazine[Title/Abstract] OR Acetazolamide[Title/Abstract] OR "sodium nitroprusside"[Title/Abstract] OR "clonidine hydrochloride"[Title/Abstract] OR "Potassium-sparing agents"[Title/Abstract] OR "loop diuretic"[Title/Abstract] OR "sodium nitroprusside"[Title/Abstract] OR moxonidine[Title/Abstract] OR "renin inhibitor"[Title/Abstract] OR "Angiotensin-Converting Enzyme Inhibitors"[MeSH:noexp] OR "angiotensin converting enzyme inhibit*"[Title/Abstract] OR alacepril[Title/Abstract] OR altiopril[Title/Abstract] OR ancovenin[Title/Abstract] OR benazepril[Title/Abstract] OR captopril[Title/Abstract] OR ceranapril[Title/Abstract] OR ceronapril[Title/Abstract] OR cilazapril[Title/Abstract] OR deacetylalacepril[Title/Abstract] OR delapril[Title/Abstract] OR derapril[Title/Abstract] OR enalapril[Title/Abstract] OR epicaptopril[Title/Abstract] OR fasidotril[Title/Abstract] OR fosinopril[Title/Abstract] OR foroxymithine[Title/Abstract] OR gemopatrilat[Title/Abstract] OR idapril[Title/Abstract] OR imidapril[Title/Abstract] OR indolapril[Title/Abstract] OR libenzapril[Title/Abstract] OR lisinopril[Title/Abstract] OR moexipril[Title/Abstract] OR moveltipril[Title/Abstract] OR omapatrilat[Title/Abstract] OR pentopril*[Title/Abstract] OR perindopril*[Title/Abstract] OR pivopril[Title/Abstract] OR quinapril*[Title/Abstract] OR ramipril*[Title/Abstract] OR rentiapril[Title/Abstract] OR saralasin[Title/Abstract] OR "s nitrosocaptopril"[Title/Abstract] OR spirapril*[Title/Abstract] OR temocapril*[Title/Abstract] OR teprotide[Title/Abstract] OR trandolapril*[Title/Abstract] OR utibapril*[Title/Abstract] OR zabicipril*[Title/Abstract] OR zofenopril*[Title/Abstract] OR Aceon[Title/Abstract] OR Accupril[Title/Abstract] OR Altace[Title/Abstract] OR Capoten[Title/Abstract] OR Lotensin[Title/Abstract] OR Mavik[Title/Abstract] OR Monopril[Title/Abstract] OR Prinivil[Title/Abstract] OR Univas[Title/Abstract] OR Vasotec[Title/Abstract] OR Zestril[Title/Abstract] OR "Angiotensin Receptor Antagonists"[MeSH:noexp] OR abitesartan[Title/Abstract] OR azilsartan[Title/Abstract] OR candesartan[Title/Abstract] OR elisartan[Title/Abstract] OR embusartan[Title/Abstract] OR eprosartan[Title/Abstract] OR forasartan[Title/Abstract] OR irbesartan[Title/Abstract] OR losartan[Title/Abstract] OR milfasartan[Title/Abstract] OR olmesartan[Title/Abstract] OR saprisartan[Title/Abstract] OR tasosartan[Title/Abstract] OR telmisartan[Title/Abstract] OR valsartan[Title/Abstract] OR zolasartan[Title/Abstract] OR Atacand[Title/Abstract] OR Avapro[Title/Abstract] OR Benicar[Title/Abstract] OR Cozaar[Title/Abstract] OR Diovan[Title/Abstract] OR Micardis[Title/Abstract] OR Teveten[Title/Abstract] OR "Calcium Channel Blockers"[MeSH:noexp] OR amlodipine[Title/Abstract] OR aranidipine[Title/Abstract] OR barnidipine[Title/Abstract] OR bencyclane[Title/Abstract] OR benidipine[Title/Abstract] OR bepridil[Title/Abstract] OR cilnidipine[Title/Abstract] OR cinnarizine[Title/Abstract] OR clentiazem[Title/Abstract] OR darodipine[Title/Abstract] OR diltiazem[Title/Abstract] OR efonidipine[Title/Abstract] OR elgodipine[Title/Abstract] OR etafenone[Title/Abstract] OR fantofarone[Title/Abstract] OR felodipine[Title/Abstract] OR fendiline[Title/Abstract] OR flunarizine[Title/Abstract] OR gallopamil[Title/Abstract] OR isradipine[Title/Abstract] OR lacidipine[Title/Abstract] OR lercanidipine[Title/Abstract] OR lidoflazine[Title/Abstract] OR lomerizine[Title/Abstract] OR manidipine[Title/Abstract] OR mibefradil[Title/Abstract] OR nicardipine[Title/Abstract] OR nifedipine[Title/Abstract] OR niguldipine[Title/Abstract] OR nilvadipine[Title/Abstract] OR nimodipine[Title/Abstract] OR nisoldipine[Title/Abstract] OR nitrendipine[Title/Abstract] OR perhexiline[Title/Abstract] OR prenylamine[Title/Abstract] OR semotiadil[Title/Abstract] OR terodiline[Title/Abstract] OR tiapamil[Title/Abstract] OR verapamil[Title/Abstract] OR Cardizem CD[Title/Abstract] OR "Dilacor XR"[Title/Abstract] OR Tiazac[Title/Abstract] OR "Cardizem Calan"[Title/Abstract] OR Isoptin[Title/Abstract] OR "Calan SR"[Title/Abstract] OR "Isoptin SR Coer"[Title/Abstract] OR "Covera HS"[Title/Abstract] OR "Verelan PM"[Title/Abstract] OR Nondihydropyridines[Title/Abstract] OR Dihydropyridines[Title/Abstract] OR "ACE inhibitors" OR ACEi OR "Angiotensin II receptor blockers" OR "angiotensin II receptor"[Title/Abstract] OR methyldopa[Title/Abstract] OR alphamethyldopa[Title/Abstract] OR amodopa[Title/Abstract] OR dopamet[Title/Abstract] OR dopegyt[Title/Abstract] OR dopegit[Title/Abstract] OR dopegite[Title/Abstract] OR emdopa[Title/Abstract] OR hyperpax[Title/Abstract] OR hyperpaxa[Title/Abstract] OR "methylpropionic acid"[Title/Abstract] OR dopergit[Title/Abstract] OR meldopa[Title/Abstract] OR methyldopate[Title/Abstract] OR medopa[Title/Abstract] OR medomet[Title/Abstract] OR sembrina[Title/Abstract] OR aldomet[Title/Abstract] OR aldometil[Title/Abstract] OR aldomin[Title/Abstract] OR hydopa[Title/Abstract] OR methyldihydroxyphenylalanine[Title/Abstract] OR "methyl dopa"[Title/Abstract] OR mulfasin[Title/Abstract] OR presinol[Title/Abstract] OR presolisin[Title/Abstract] OR sedometil[Title/Abstract] OR taquinil[Title/Abstract] OR dihydroxyphenylalanine[Title/Abstract] OR methylphenylalanine[Title/Abstract] OR methylalanine[Title/Abstract] OR "alpha methyl dopa"[Title/Abstract] OR reserpine[Title/Abstract] OR serpentina[Title/Abstract] OR rauwolfia[Title/Abstract] OR serpasil[Title/Abstract] OR clonidine[Title/Abstract] OR adesipress[Title/Abstract] OR arkamin[Title/Abstract] OR caprysin[Title/Abstract] OR catapres*[Title/Abstract] OR catasan[Title/Abstract] OR chlofazolin[Title/Abstract] OR chlophazolin[Title/Abstract] OR clinidine[Title/Abstract] OR clofelin*[Title/Abstract] OR clofenil[Title/Abstract] OR clomidine[Title/Abstract] OR clondine[Title/Abstract] OR clonistada[Title/Abstract] OR clonnirit[Title/Abstract] OR clophelin*[Title/Abstract] OR dichlorophenylaminoimidazoline[Title/Abstract] OR dixarit[Title/Abstract] OR duraclon[Title/Abstract] OR gemiton[Title/Abstract] OR haemiton[Title/Abstract] OR hemiton[Title/Abstract] OR imidazoline[Title/Abstract] OR isoglaucon[Title/Abstract] OR klofelin[Title/Abstract] OR klofenil[Title/Abstract] OR normopresan[Title/Abstract] OR paracefan[Title/Abstract] OR "tesno timelets"[Title/Abstract] OR Hydralazine[MeSH:noexp] OR hydralazin*[Title/Abstract] OR hydrallazin*[Title/Abstract] OR hydralizine[Title/Abstract] OR hydrazinophtalazine[Title/Abstract] OR hydrazinophthalazine[Title/Abstract] OR hydrazinophtalizine[Title/Abstract] OR dralzine[Title/Abstract] OR hydralacin[Title/Abstract] OR hydrolazine[Title/Abstract] OR hypophthalin[Title/Abstract] OR hypoftalin[Title/Abstract] OR idralazina[Title/Abstract] OR "1-hydrazinophthalazine"[Title/Abstract] OR apressin[Title/Abstract] OR nepresol[Title/Abstract] OR apressoline[Title/Abstract] OR apresoline[Title/Abstract] OR apresolin[Title/Abstract] OR alphapress[Title/Abstract] OR alazine[Title/Abstract] OR lopress[Title/Abstract] OR plethorit[Title/Abstract] OR praeparat[Title/Abstract] OR "Adrenergic beta-Antagonists"[MeSH:noexp] OR acebutolol[Title/Abstract] OR adimolol[Title/Abstract] OR afurolol[Title/Abstract] OR alprenolol[Title/Abstract] OR amosulalol[Title/Abstract] OR arotinolol[Title/Abstract] OR atenolol[Title/Abstract] OR befunolol[Title/Abstract] OR betaxolol[Title/Abstract] OR bevantolol[Title/Abstract] OR bisoprolol[Title/Abstract] OR bopindolol[Title/Abstract] OR bornaprolol[Title/Abstract] OR brefonalol[Title/Abstract] OR bucindolol[Title/Abstract] OR bucumolol[Title/Abstract] OR bufetolol[Title/Abstract] OR bufuralol[Title/Abstract] OR bunitrolol[Title/Abstract] OR bunolol[Title/Abstract] OR bupranolol[Title/Abstract] OR butofilolol[Title/Abstract] OR butoxamine[Title/Abstract] OR carazolol[Title/Abstract] OR carteolol[Title/Abstract] OR carvedilol[Title/Abstract] OR celiprolol[Title/Abstract] OR cetamolol[Title/Abstract] OR "chlortalidone cloranolol"[Title/Abstract] OR cyanoiodopindolol[Title/Abstract] OR cyanopindolol[Title/Abstract] OR deacetylmetipranolol[Title/Abstract] OR diacetolol[Title/Abstract] OR dihydroalprenolol[Title/Abstract] OR dilevalol[Title/Abstract] OR epanolol[Title/Abstract] OR esmolol[Title/Abstract] OR exaprolol[Title/Abstract] OR falintolol[Title/Abstract] OR flestolol[Title/Abstract] OR flusoxolol[Title/Abstract] OR hydroxybenzylpinodolol[Title/Abstract] OR hydroxycarteolol[Title/Abstract] OR hydroxymetoprolol[Title/Abstract] OR indenolol[Title/Abstract] OR iodocyanopindolol[Title/Abstract] OR iodopindolol[Title/Abstract] OR iprocrolol[Title/Abstract] OR isoxaprolol[Title/Abstract] OR labetalol[Title/Abstract] OR landiolol[Title/Abstract] OR levobunolol[Title/Abstract] OR levomoprolol[Title/Abstract] OR medroxalol[Title/Abstract] OR mepindolol[Title/Abstract] OR methylthiopropranolol[Title/Abstract] OR metipranolol[Title/Abstract] OR metoprolol[Title/Abstract] OR moprolol[Title/Abstract] OR nadolol[Title/Abstract] OR oxprenolol[Title/Abstract] OR penbutolol[Title/Abstract] OR pindolol[Title/Abstract] OR nebivolol[Title/Abstract] OR nifenalol[Title/Abstract] OR nipradilol[Title/Abstract] OR pafenolol[Title/Abstract] OR pamatolol[Title/Abstract] OR practolol[Title/Abstract] OR primidolol[Title/Abstract] OR prizidilol[Title/Abstract] OR procinolol[Title/Abstract] OR pronetalol[Title/Abstract] OR propranolol[Title/Abstract] OR proxodolol[Title/Abstract] OR ridazolol[Title/Abstract] OR salcardolol[Title/Abstract] OR soquinolol[Title/Abstract] OR sotalol[Title/Abstract] OR spirendolol[Title/Abstract] OR talinolol[Title/Abstract] OR tertatolol[Title/Abstract] OR tienoxolol[Title/Abstract] OR tilisolol[Title/Abstract] OR timolol[Title/Abstract] OR tolamolol[Title/Abstract] OR toliprolol[Title/Abstract] OR tribendilol[Title/Abstract] OR xibenolol[Title/Abstract] OR "Adrenergic alpha-Antagonists"[MeSH:noexp] OR alfuzosin[Title/Abstract] OR bunazosin[Title/Abstract] OR doxazosin[Title/Abstract] OR metazosin[Title/Abstract] OR neldazosin[Title/Abstract] OR prazosin[Title/Abstract] OR silodosin[Title/Abstract] OR tamsulosin[Title/Abstract] OR terazosin[Title/Abstract] OR tiodazosin[Title/Abstract] OR trimazosin[Title/Abstract] OR urapidil[Title/Abstract] OR "beta-adrenoceptor blocking drugs"[Title/Abstract] OR "vasodilator antihypertensive drugs"[Title/Abstract] OR "centrally acting antihypertensive drugs"[Title/Abstract] OR "Adrenergic alpha-Antagonists"[Title/Abstract] OR "adrenergic receptor antagonist"[Title/Abstract] OR "adrenergic receptor blocker"[Title/Abstract] OR "alpha-2 adrenergic receptor agonist"[Title/Abstract])) AND ((humans[Filter]) AND (english[Filter]))) AND ((("Neurodevelopmental Disorders"[MeSH] OR "Cognition Disorders"[MeSH:noexp] OR "Neurocognitive Disorders"[MeSH:noexp] OR "Autism Spectrum Disorder"[MeSH] OR "Attention Deficit Disorder with Hyperactivity"[MeSH:noexp] OR "Motor Disorders"[MeSH:noexp] OR Epilepsy[MeSH:noexp] OR "Intellectual Disability"[MeSH:noexp] OR "congenital abnormalities"[MeSH] OR "congenital defect*"[Title/Abstract] OR "congenital malformation*"[Title/Abstract] OR "congenital anomal*"[Title/Abstract] OR "longer term outcome*"[Title/Abstract] OR "long-term outcome*"[Title/Abstract] OR "long term outcome*"[Title/Abstract] OR "Child Development"[MeSH:noexp] OR "Executive Function"[MeSH:noexp] OR "executive function*"[Title/Abstract] OR "child development*"[Title/Abstract] OR Neuropsychology[MeSH:noexp] OR neurodevelopment*[Title/Abstract] OR "neurodevelopmental disorder*"[Title/Abstract] OR "educational difficult*"[Title/Abstract] OR "mental deficienc*"[Title/Abstract] OR "cognitive deficit*"[Title/Abstract] OR "poor performance"[Title/Abstract] OR IQ[Title/Abstract] OR "intelligence quotient"[Title/Abstract] OR Behavio*[Title/Abstract] OR "behavioural disturbance*"[Title/Abstract] OR social*[Title/Abstract] OR emotion*[Title/Abstract] OR "motor impairment*"[Title/Abstract] OR communication*[Title/Abstract] OR "learning disabilit*"[Title/Abstract] OR "learning difficult*"[Title/Abstract] OR "learning disorder*"[Title/Abstract] OR autism[Title/Abstract] OR "intellectual disabilit*"[Title/Abstract] OR "intellectual impairment*"[Title/Abstract] OR "mental retardation*"[Title/Abstract] OR "attention deficit hyperactivity disorder*"[Title/Abstract] OR "attention deficit disorder*"[Title/Abstract] OR ADHD[Title/Abstract] OR epilepsy[Title/Abstract] OR "cerebral palsy"[Title/Abstract] OR "neurocognitive disorder*"[Title/Abstract] OR "cognitive impairment*"[Title/Abstract] OR "cognitive disabilit*"[Title/Abstract] OR "developmental disorder*"[Title/Abstract] OR "developmental disabilit*"[Title/Abstract] OR "development disabilit*"[Title/Abstract] OR "cognitive disturbance*"[Title/Abstract] OR "hypertensive pregnancy disorder*"[Title/Abstract] OR "hypertensive disorders of pregnancy"[Title/Abstract] OR "autism spectrum"[Title/Abstract] OR autistic[Title/Abstract] OR "autism spectrum disorder*"[Title/Abstract] OR Asperger[Title/Abstract] OR "Asperger's"[Title/Abstract] OR "Asperger's Syndrome"[Title/Abstract] OR hyperactive*[Title/Abstract] OR overactive*[Title/Abstract] OR inattention[Title/Abstract] OR "hyperkinetic disorder*"[Title/Abstract] OR hyperkinet*[Title/Abstract] OR neurodevelopment[Title/Abstract] OR "communication disorder*"[Title/Abstract] OR "motor disorder*"[Title/Abstract] OR "school performance"[Title/Abstract])) AND ((humans[Filter]) AND (english[Filter]))) AND (((Infant[MeSH] OR child[MeSH] OR baby[Title/Abstract] OR babies[Title/Abstract] OR offspring*[Title/Abstract] OR infant*[Title/Abstract] OR toddler*[Title/Abstract] OR preschool*[Title/Abstract] OR child*[Title/Abstract] OR kindergarten*[Title/Abstract] OR "young person"[Title/Abstract] OR youth[Title/Abstract] OR fetal[Title/Abstract] OR foetal[Title/Abstract] OR fetus[Title/Abstract] OR foetus[Title/Abstract] OR prenatal[Title/Abstract] OR pre school[Title/Abstract] OR pre-school[Title/Abstract])) AND ((humans[Filter]) AND (english[Filter])))

**Database (4): Web of Science – 04/07/2025**

Search: TS=(pregnan* or prenatal or perinatal or maternal)

Search: TS=(gestational AND hypertens*)

Search: TS=(gestational NEAR hypertens*)

Search: TS=(pre-eclamp* or preeclamp* or eclamp* or toxemia or toxaemia or "Maternal hypertens*")

Search: TS=(hypertens* NEAR pregnan*)

Search: TS=(hypertens* AND pregnan*)

Search: TS=Toxemia

Search: TS=Eclampsia

Search: TS=Pre-Eclampsia

Search: TS="Pregnancy Complications, Cardiovascular"

Search: TS="Hypertension, Pregnancy-Induced"

Search: TS=pregnancy

Search: #1 OR #2 OR #3 OR #4 OR #5 OR #6 OR #7 OR #8 OR #9 OR #10 OR #11 OR #12

Search: TS="Antihypertensive Agents"

Search: TS=Thiazides

Search: TS="Sodium Chloride Symporter Inhibitors"

Search: TS="Sodium Potassium Chloride Symporter Inhibitors"

Search: TS=(amiloride or benzothiadiazine or bendroflumethiazide or bumetanide or chlorothiazide or cyclopenthiazide or furosemide or hydrochlorothiazide or hydroflumethiazide or methyclothiazide or metolazone or polythiazide or trichlormethiazide or veratide or thiazide*)

Search: TS=(chlorthalidone or chlortalidone or phthalamudine or chlorphthalidolone or oxodoline or thalitone or hygroton or indapamide or metindamide)

Search: TS=(Dihydralazine or Acetazolamide or "sodium nitroprusside" or "clonidine hydrochloride" or "Potassium-sparing agents" or "loop diuretic" or "sodium nitroprusside" or moxonidine or "renin inhibitor")

Search: #15 OR #16 OR #17 OR #18 OR #19 OR #20

Search: TS="Angiotensin-Converting Enzyme Inhibitors"

Search: TS="angiotensin converting enzyme inhibit*"

Search: TS=(alacepril or altiopril or ancovenin or benazepril or captopril or ceranapril or ceronapril or cilazapril or deacetylalacepril or delapril or derapril or enalapril or epicaptopril or fasidotril or fosinopril or foroxymithine or gemopatrilat or idapril or imidapril or indolapril or libenzapril or lisinopril or moexipril or moveltipril or omapatrilat or pentopril* or perindopril* or pivopril or quinapril* or ramipril* or rentiapril or saralasin or s nitrosocaptopril or spirapril* or temocapril* or teprotide or trandolapril* or utibapril* or zabicipril* or zofenopril* or Aceon or Accupril or Altace or

Capoten or Lotensin or Mavik or Monopril or Prinivil or Univas or Vasotec or Zestril)

Search: #22 OR #23 OR #24

Search: TS="Angiotensin Receptor Antagonists"

Search: TS=(abitesartan or azilsartan or candesartan or elisartan or embusartan or eprosartan or forasartan or irbesartan or losartan or milfasartan or olmesartan or saprisartan or tasosartan or telmisartan or valsartan or zolasartan or Atacand or Avapro or Benicar or Cozaar or Diovan or Micardis or Teveten)

Search: #26 OR #27

Search: TS="Calcium Channel Blockers"

Search: TS=(amlodipine or aranidipine or barnidipine or bencyclane or benidipine or bepridil or

cilnidipine or cinnarizine or clentiazem or darodipine or diltiazem or efonidipine or elgodipine or etafenone or fantofarone or felodipine or fendiline or flunarizine or gallopamil or isradipine or lacidipine or lercanidipine or lidoflazine or lomerizine or manidipine or mibefradil or nicardipine or nifedipine or niguldipine or nilvadipine or nimodipine or nisoldipine or nitrendipine or perhexiline or prenylamine or semotiadil or terodiline or tiapamil or verapamil or "Cardizem CD" or "Dilacor XR" or Tiazac or "Cardizem Calan" or Isoptin or "Calan SR" or "Isoptin SR Coer" or "Covera HS" or "Verelan PM")

Search: TS=(Nondihydropyridines or Dihydropyridines or "ACE inhibitors" or ACEi or "Angiotensin II receptor blockers" or "angiotensin II receptor")

Search: #29 OR #30 OR #31

Search: TS=(methyldopa or alphamethyldopa or amodopa or dopamet or dopegyt or dopegit or

dopegite or emdopa or hyperpax or hyperpaxa or methylpropionic acid or dopergit or meldopa or methyldopate or medopa or medomet or sembrina or aldomet or aldometil or aldomin or hydopa or methyldihydroxyphenylalanine or "methyl dopa" or mulfasin or presinol or presolisin or sedometil or sembrina or taquinil or dihydroxyphenylalanine or methylphenylalanine or methylalanine or "alpha methyl dopa")

Search: TS=(reserpine or serpentina or rauwolfia or serpasil)

Search: TS=(clonidine or adesipress or arkamin or caprysin or catapres* or catasan or chlofazolin or chlophazolin or clinidine or clofelin* or clofenil or clomidine or clondine or clonistada or clonnirit or clophelin* or dichlorophenylaminoimidazoline or dixarit or duraclon or gemiton or haemiton or hemiton or imidazoline or isoglaucon or klofelin or klofenil or normopresan or paracefan or "tesno timelets")

Search: TS=Hydralazine

Search: TS=(hydralazin* or hydrallazin* or hydralizine or hydrazinophtalazine or

hydrazinophthalazine or hydrazinophtalizine or dralzine or hydralacin or hydrolazine or hypophthalin or hypoftalin or hydrazinophthalazine or idralazina or 1-hydrazinophthalazine or apressin or nepresol or apressoline or apresoline or apresolin or alphapress or alazine or idralazina or lopress or plethorit or praeparat)

Search: #33 OR #34 OR #35 OR #36 OR #37

Search: TS="Adrenergic beta-Antagonists"

Search: TS=(acebutolol or adimolol or afurolol or alprenolol or amosulalol or arotinolol or atenolol or befunolol or betaxolol or bevantolol or bisoprolol or bopindolol or bornaprolol or brefonalol or bucindolol or bucumolol or bufetolol or bufuralol or bunitrolol or bunolol or bupranolol or butofilolol or butoxamine or carazolol or carteolol or carvedilol or celiprolol or cetamolol or chlortalidone cloranolol or cyanoiodopindolol or cyanopindolol or deacetylmetipranolol or diacetolol or dihydroalprenolol or dilevalol or epanolol or esmolol or exaprolol or falintolol or

flestolol or flusoxolol or hydroxybenzylpinodolol or hydroxycarteolol or hydroxymetoprolol or indenolol or iodocyanopindolol or iodopindolol or iprocrolol or isoxaprolol or labetalol or landiolol or levobunolol or levomoprolol or medroxalol or mepindolol or methylthiopropranolol or metipranolol or metoprolol or moprolol or nadolol or oxprenolol or penbutolol or pindolol or nadolol or nebivolol or nifenalol or nipradilol or oxprenolol or pafenolol or pamatolol or penbutolol or pindolol or practolol or primidolol or prizidilol or procinolol or pronetalol or propranolol or proxodolol or ridazolol or salcardolol or soquinolol or sotalol or spirendolol or talinolol or tertatolol or tienoxolol or tilisolol or timolol or tolamolol or toliprolol or tribendilol or xibenolol)

Search: TS="Adrenergic alpha-Antagonists"

Search: TS=(alfuzosin or bunazosin or doxazosin or metazosin or neldazosin or prazosin or silodosin or tamsulosin or terazosin or tiodazosin or trimazosin or urapidil)

Search: TS=("beta-adrenoceptor blocking drugs" or "vasodilator antihypertensive drugs" or "centrally acting antihypertensive drugs" or "Adrenergic alpha-Antagonists" or "adrenergic receptor antagonist" or "adrenergic receptor blocker" or "alpha-2 adrenergic receptor agonist")

Search: #39 OR #40 OR #41 OR #42 OR #43

Search: #14 OR #21 OR #25 OR #28 OR #32 OR #38 OR #44

Search: TS=infant

Search: TS=child

Search: TS=(Baby or babies or offspring* or infant* or toddler* or preschool* or child* or kindergarten* or "young person" or youth or fetal or foetal or fetus or foetus or prenatal or "pre school" or pre-school)

Search: #46 OR #47 OR #48

Search: TS= (congenital NEAR anomal*)

Search: TS= (congenital NEAR malformation*)

Search: TS=(congenital NEAR defect*)

Search: TS="Congenital Abnormalities"

Search: TS=(neurodevelopment* or "neurodevelopmental disorder*" or "educational difficult*" or "mental deficienc*" or "cognitive deficit*" or "poor performance" or IQ or "intelligence quotient" or behavio* or "behavioural disturbance*" or social* or emotion* or "motor impairment*" or communication* or "learning disabilit*" or "learning difficult*" or "learning disorder*" or autism or "intellectual disabilit*" or "intellectual impairment*" or "mental retardation*" or "attention deficit hyperactivity disorder*" or "attention deficit disorder*" or ADHD or epilepsy or "cerebral palsy" or "neurocognitive disorder*" or "cognitive impairment*" or "cognitive disabilit*" or "developmental disorder*" or "developmental disabilit*" or "development disabilit*" or "cognitive disturbance*" or "hypertensive pregnancy disorder*" or "hypertensive disorders of pregnancy" or "autism spectrum" or autistic or "autism spectrum disorder*" or Asperger or Asperger's or "Asperger's Syndrome" or hyperactive* or overactive* or inattention or "hyperkinetic disorder*" or hyperkinet* or neurodevelopment or "communication disorder*" or "motor disorder*" or "school performance")

Search: TS=neuropsycholog*

Search: TS=Neuropsychology

Search: TS="Cognitive Dysfunction"

Search: TS="Executive Function*"

Search: TS="Executive Function"

Search: TS="Child development*"

Search: TS="Child Development"

Search: TS=("longer term outcome" or "long term outcome*" or "long-term outcome*")

Search: TS="Intellectual Disability"

Search: TS=Epilepsy

Search: TS="Motor Disorders"

Search: TS="Attention Deficit Disorder with Hyperactivity"

Search: TS="Autism Spectrum Disorder"

Search: TS="Neurocognitive Disorders"

Search: TS="Cognition Disorders"

Search: TS="Neurodevelopmental Disorders"

Search: #50 OR #51 OR #52 OR #53 OR #54 OR #55 OR #56 OR #57 OR #58 OR #59 OR #60 OR #61 OR #62 OR #63 OR #64 OR #65 OR #66 OR #67 OR #68 OR #69 OR #70

Search: #45 AND #49 AND #71 AND #13 and English (Languages) and Humans
